# Securing Optical Networks Using Quantum-Secured Blockchain: An Overview

**DOI:** 10.3390/s23031228

**Published:** 2023-01-20

**Authors:** Purva Sharma, Kwonhue Choi, Ondrej Krejcar, Pavel Blazek, Vimal Bhatia, Shashi Prakash

**Affiliations:** 1 Signals and Software Group, Department of Electrical Engineering, Indian Institute of Technology Indore, Indore 453552, India; 2 Department of Information and Communication Engineering, Yeungnam University, Gyeongsan 38541, Republic of Korea; 3 Center for Basic and Applied Research, Faculty of Informatics and Management, University of Hradec Kralove, 500 03 Hradec Kralove, Czech Republic; 4 Institute of Technology and Business in Ceske Budejovice, 370 01 Ceske Budejovice, Czech Republic; 5 Malaysia Japan International Institute of Technology (MJIIT), University Teknologi Malaysia, Kuala Lumpur 54100, Malaysia; 6 Photonics Laboratory, Department of Electronics and Instrumentation Engineering, Institute of Engineering and Technology, Devi Ahilya University, Indore 452017, India

**Keywords:** quantum key distribution, blockchain, quantum-secured blockchain, optical networks, attacks, security

## Abstract

The deployment of optical network infrastructure and development of new network services are growing rapidly for beyond 5/6G networks. However, optical networks are vulnerable to several types of security threats, such as single-point failure, wormhole attacks, and Sybil attacks. Since the uptake of e-commerce and e-services has seen an unprecedented surge in recent years, especially during the COVID-19 pandemic, the security of these transactions is essential. Blockchain is one of the most promising solutions because of its decentralized and distributed ledger technology, and has been employed to protect these transactions against such attacks. However, the security of blockchain relies on the computational complexity of certain mathematical functions, and because of the evolution of quantum computers, its security may be breached in real-time in the near future. Therefore, researchers are focusing on combining quantum key distribution (QKD) with blockchain to enhance blockchain network security. This new technology is known as quantum-secured blockchain. This article describes different attacks in optical networks and provides a solution to protect networks against security attacks by employing quantum-secured blockchain in optical networks. It provides a brief overview of blockchain technology with its security loopholes, and focuses on QKD, which makes blockchain technology more robust against quantum attacks. Next, the article provides a broad view of quantum-secured blockchain technology. It presents the network architecture for the future research and development of secure and trusted optical networks using quantum-secured blockchain. The article also highlights some research challenges and opportunities.

## 1. Introduction

Optical network infrastructure and services are rapidly growing because of ever-increasing bandwidth-hungry applications such as cloud computing, video conferencing, video messaging, and others. However, optical networks are vulnerable to various types of security breaches, such as service disruption attacks and physical infrastructure attacks [1,2]. Service disruption attacks degrade the performance by inserting interfering signals in the channel for jamming and alien-wavelength attacks. Physical infrastructure attacks, including single component failure, disaster attacks, and critical location attacks, physically damage the optical network infrastructure, such as links or node failure. Currently, in the control plane, software-defined network (SDN) [3,4,5,6,7,8] controllers are installed, which provide logically centralized control to network operators and efficiently manage the network resources. However, an SDN controller is prone to single-point failure, thereby making optical networks insecure. A malicious attacker may also use wormhole attacks and Sybil attacks to disable networks by creating fake network resources [9]. The increase in security attacks such as single point failure, wormhole attacks [10], and Sybil attacks [11] makes optical networks insecure and unreliable [1], and can cause huge data and revenue losses [2]. Hence, securing optical networks against various attacks is paramount. Recently, blockchain technology has been incorporated into optical network architecture to build trust between untrusted nodes in the network by monitoring the network resources in a distributed manner [9]. Therefore, blockchain technology [12,13,14,15,16,17,18,19,20,21,22] is used in optical networks to avoid such types of attacks by creating a decentralized environment [9,23].

A blockchain is a distributed ledger or database based on cryptographic protection against malicious attacks [24]. This technique allows users to share information among nodes in the network that do not trust each other [12]. The attractive features of blockchain, such as transparency, privacy, and accountability [25], make it reliable for a variety of applications related to secure communication [9,26], smart contracts [27], healthcare [28,29,30], supply chain management [31,32], industries [33], and other financial services. Blockchain technology came to the mainstream with its most prominent application, namely the cryptocurrency Bitcoin [34,35,36]. It is estimated that 10% of global gross domestic product (GDP) will be saved on blockchain technology by 2025 [37]; hence, blockchain technology has received extensive research attention.

In software-defined optical networks (SDONs), the blockchain technology was introduced to provide trusted multi-controller routing to implement efficient failure recovery mechanisms and to ratify the quality of transmission (QoT) performance [38]. In [38,39], a novel framework based on blockchain technology was proposed to provide trusted service level agreement (SLA) accounting in optical networks. In [40], a new architecture was presented in multi-domain scenarios to manage the network resources using blockchain without needing a single centralized authority. Blockchain-assisted spectrum trading was also proposed in [41] to achieve the security of trading records between virtual optical networks (VONs). Furthermore, a blockchain-based BlockONet architecture has been presented to secure access identification for 5G fronthaul [42].

Blockchain security is based on one-way mathematical functions and cryptographic algorithms, which are hard to hack. Conventional computers take many years to break blockchain security. However, with the commercialization of quantum computers in the near future [43,44], blockchain security can be compromised [45] in real-time. Thus, in order to improve the security of blockchain, post-quantum cryptography schemes have been designed [46]. However, currently, such schemes are not strong enough and efficient enough to guarantee security against quantum attacks, and they are still in their infancy. Therefore, there has been a renewed interest in research on enhancing the security of blockchain using quantum technologies.

The security of quantum communication relies on fundamental principles of quantum mechanics, i.e., the Heisenberg uncertainty principle and quantum no-cloning theorem [47,48,49]. The uncertainty principle states that it is not possible to simultaneously measure the position and momentum of particles such as photons [50,51]. Furthermore, any arbitrary unknown quantum state cannot be copied as stated by the quantum no-cloning theorem [52,53]. Quantum key distribution (QKD) [54,55,56,57,58,59,60] is one of the most prominent applications of quantum communication. QKD generates and distributes secret keys between the end-users to encrypt and decrypt confidential information [47,49,58,61]. The secret key information is transmitted through a quantum signal channel (QSCh); hence, either the sender or receiver can easily detect any security attack. Hence, QKD has the potential to improve the security of a blockchain network. Therefore, integrating QKD with blockchain is envisaged to pave the way for a new and secure technology termed quantum-secured blockchain [45,62]. Thus, this new and secure technology is a promising solution for improving the security and performance of optical networks against malicious attacks.

### 1.1. Related Work

The integration of QKD and blockchain technology opens a new era that increases the security of the overall network/system. In [45], a prototype of a quantum-safe blockchain platform was developed that uses the QKD network to establish secure authentication in blockchain against quantum computing attacks. Additionally, Ref. [45] selects a broadcast protocol, where all nodes agree on new blocks under equal circumstances, rather than giving the control of creating new blocks to a single miner. A framework of a quantum-secured and permissioned blockchain, namely Logicontract, was proposed in [63]. This system uses a voting-based consensus protocol and a QKD-based digital signature scheme to achieve consensus on the blockchain. In [64], entanglement in time was used in a conceptual design for a quantum-secured blockchain [65,66,67]. In this approach, blockchain was encoded into a temporal Greenberger–Horne–Zeilinger (GHZ) state of photons that do not coexist at the same time, which gives the essential quantum advantage. In [68], a multiscale technique and quantum and relativistic mechanics were used to solve the democracy and randomness of block verification, as well as the assignment of the new blocks. A simple hybrid classical–quantum payment system was constructed by combining a classical blockchain and quantum lightning in [69]. In a public-key quantum money system, they used quantum states as banknotes and utilized blockchain to solve the trust issue with quantum banknotes. A new quantum-inspired quantum-walk-based authentication and encryption protocol was presented to build a blockchain framework for secure data transmission among IoT devices in [70]. A new quantum blockchain scheme using quantum entanglement and delegated proof of stake (DPoS) was proposed and examined against several attacks, such as double spending attack, man-in-the-middle attack, and state estimation attack [71]. Based on security concerns, a new distributed quantum electronic medical record system and a new private quantum blockchain network were proposed in [72]. However, the research in the field of quantum blockchain is still in the initial phase of development.

Inspired by the above studies, this article aims to cover the overview of blockchain technology and one of the most promising applications of quantum communication, i.e., QKD with their underlying process. In addition, the article describes the motivation behind integrating QKD with blockchain and explains the process of secure data transmission using quantum-secured blockchain. Moreover, in order to prevent optical networks against security breaches, a distributed quantum-secured blockchain optical network architecture is presented. Furthermore, some of the most relevant challenges and research aspects related to quantum-secured blockchain are highlighted in this article.

### 1.2. Contribution of This Article

The main contributions of this article are as follows:

1. This article briefly discusses the attacks in optical networks and provides a concise overview of blockchain technology, including the process of blockchain, the working of blockchain, features of blockchain, and types of blockchain.
2. The article also discusses the concept of QKD and explains the process of secret key generation using the Bennett and Brassard-84 (BB84) QKD protocol.
3. The motivation behind integrating QKD with blockchain to form a quantum-secured blockchain and its underlying process are explained in detail.
4. The article then focuses on securing optical networks against threats using quantum-secured blockchain.
5. Finally, the article presents a distributed quantum-secured blockchain optical network architecture and provides some challenges and opportunities for future research.

### 1.3. Organization of This Article

In Section 2, an overview of blockchain technology along with its process, working, features, and types are discussed in detail. Section 3 provides a concise overview of QKD and explains the process of secret key generation using the BB84 QKD protocol. The motivation behind integrating QKD with blockchain and the process of quantum-secured blockchain technology is described in Section 4. Section 5 discusses the distributed quantum-secured blockchain optical network architecture for security. The research challenges and opportunities, along with research directions, are discussed in Section 6. Section 7 concludes the article.

## 2. Overview of Blockchain

This section gives an overview of blockchain, including the process of blockchain, the workflow of blockchain, features of blockchain, and types of blockchain.

Blockchain is an innovative and unique technology for transferring and sharing confidential information among untrusted nodes in the network. It is a distributed database that consists of non-erasable records of information [24]. In blockchain, the records are managed by a group of network nodes, not by a single centralized authority. Hence, it is tamper-resistant [73,74]. Blockchain security is based on two cryptographic tasks, i.e., a cryptographic hash function for encryption and a digital signature for authentication, which makes blockchain more secure [45]. In blockchain [34], each block is connected with its previous block using the previous block’s hash value. In addition, each node in the blockchain network has a copy of the ledger. Hence, if an eavesdropper wants to break the security of a blockchain, he/she has to solve a large mathematical problem of each node in the network at the same time, which is expensive and requires more computational power [31]. Hence, the security of blockchain technology is currently almost unbreakable. In this section, we provide a short review of blockchain and its types [12].

### 2.1. Process of Blockchain

#### 2.1.1. Blockchain Components

A blockchain consists of the following components for sharing and transferring confidential data between the end-users in the network.

*(a) Nodes*

A node is a user or a computer that requests a transaction within the blockchain networks. There are mainly two types of nodes in the blockchain networks, i.e., miner nodes and normal nodes [75]. Miner nodes validate, authenticate, and verify the new blocks using consensus protocols in the network. Such miner nodes are block generator nodes, which generate and add a new block to the blockchain ledger. Normal nodes have complete information on the blockchain content to maintain their database and cooperate with miners in the blockchain network.

*(b) Transaction*

A transaction in a blockchain network can be financial data or confidential information, depending on different applications.

*(c) Block*

A block in a blockchain is like a record book. Each block consists of data (valid transactions), a hash value of the block, a hash value of the previous block, and a timestamp.

*(d) Merkle tree root hash*

The Merkle tree root hash value is the combination of repeating hash values of individual transactions, which are hashed repeatedly until a single hash value of a block is obtained.

*(e) Block hash*

The block hash is a unique identity of a block, like a fingerprint. Once a block is created, its hash value is calculated using hashing algorithms. It is beneficial when nodes in the network want to detect some changes in the block.

*(f) Previous block hash*

The previous block’s hash value is always added to the current block’s hash to create a chain and ensure the immutability of the ledger.

*(g) Timestamp*

A timestamp includes the creation time of the block and monitors the creation time and update time of a block.

*(h) Genesis block*

A genesis block is the initial block of the blockchain. Each block in the blockchain is sequentially added to the genesis block. This block is also known as *block zero*.

*(i) Consensus protocol*

The consensus protocol is a set of rules and regulations that helps in validating a new block. Different types of consensus protocols [76] have been designed for block validation. The most widely used consensus protocols are proof-of-work (PoW), Byzantine fault tolerance (BFT), and proof-of-stake (PoS), discussed in [73].

#### 2.1.2. Working of Blockchain

**Figure 1:**
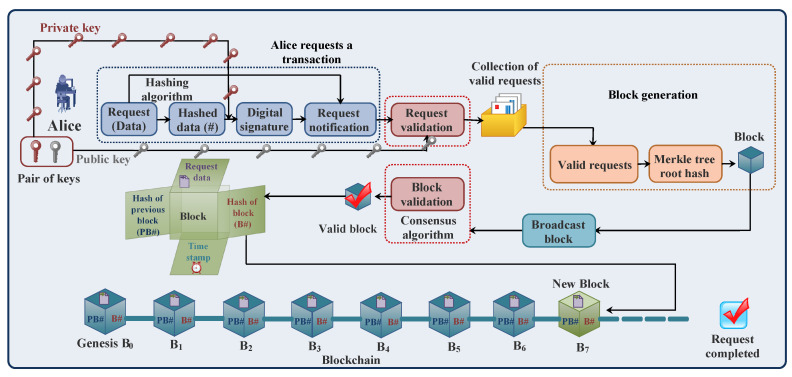
Process of blockchain.

Figure 1 describes the blockchain process. The following steps explain the working of the blockchain technology [12]:

*(a) Transaction creation*

Alice (sender) requests a transaction. Before transmission, Alice uses cryptographic algorithms to encrypt and authenticate transaction data. Alice first hashes the transaction data using hashing algorithms for data security. Each user (node) in the blockchain network generates a pair of keys, i.e., a private key and public key, using asymmetric cryptography. Alice uses her private key to sign the hashed data and generate a digital signature for authentication using elliptic curve cryptography. A public key is used by the other nodes of blockchain network to authenticate the transaction data.

*(b) Broadcast and validation of transaction*

After cryptography, both the transaction data and digital signature are broadcasted in a blockchain network. The nodes in a blockchain network validate the transaction by first decrypting the digital signature using the sender’s public key for authentication and comparing the decrypted digital signature with hashed transaction data for integrity. Then the valid transactions are collected in a block.

*(c) Broadcast and validation of block*

A block with valid transactions is broadcasted to selected miners in the network to generate a valid block. The miner uses consensus protocols to validate the block. After validation, the miner broadcasts a valid block in the blockchain network, and then the block is added to the blockchain. At the end, the ledger of each node is updated in the blockchain network, as shown in Figure 1. In this way, the request is completed.

### 2.2. Features of Blockchain

A blockchain has the following characteristics [12] that make it attractive for various types of applications.

#### 2.2.1. Decentralization

**Figure 2:**
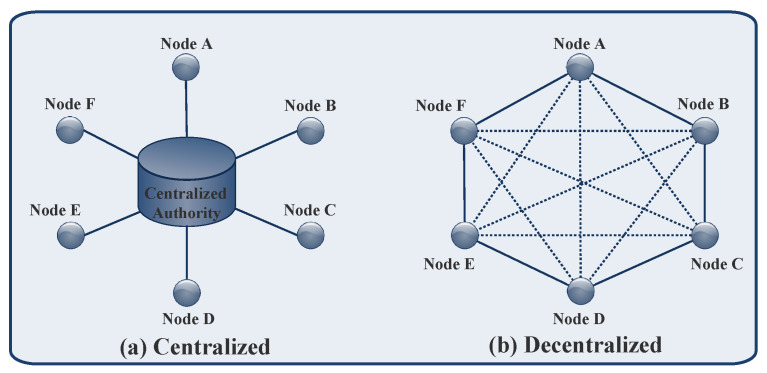
Centralized and decentralized framework.

Blockchain technology has a decentralized structure [12,77], where there is no central node/authority to store data. Moreover, in blockchain technology, transactions are not validated and authorized by a centralized authority as in a centralized system. Figure 2 depicts the framework of a centralized and decentralized system. The benefits of this feature are that: (1) it provides a trustless environment, thereby reducing maintenance costs. However, the participation of centralized authority requires maintenance costs and creates performance issues, and (2) a real-time, shared view of the data is available to all entities. Furthermore, blockchain technology employs cryptographic algorithms to maintain records and authentication in a distributed environment.

#### 2.2.2. Immutability

**Figure 3:**
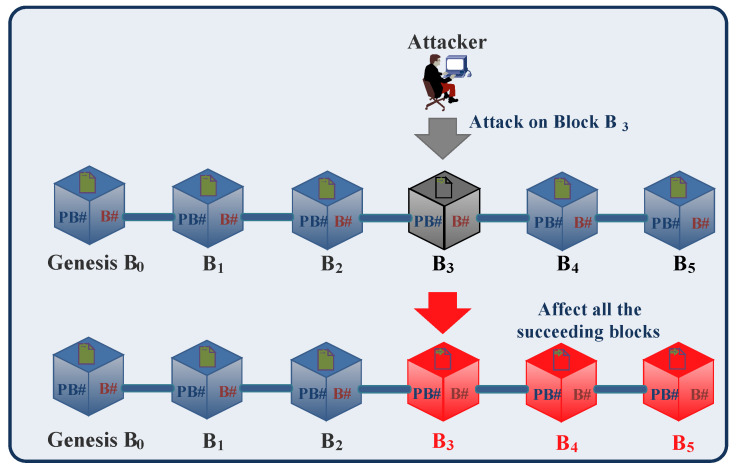
Immutability.

The blockchain is immutable [78], i.e., the previously stored data cannot be changed. All of the valid transactions are immutably stored in blocks of blockchain. In blockchain, each block is connected with the previous block using its hash value generated by a cryptographic hash function. If an attacker tries to alter any previous block record, it will affect all of the succeeding blocks of the blockchain, and the attack can be easily detectable, as shown in Figure 3. For example, in Figure 3, an attacker tries to change the records of block B_3_, and because of this feature, the changes in block B_3_ affect all of the succeeding blocks, i.e., B_4_ and B_5_ of the blockchain. Immutability ensures the security of blockchain transactions and makes data less vulnerable to attacks.

#### 2.2.3. Transparency

The blockchain system itself validates and authenticates transactions. Hence, it is transparent in recording new data and also in updating them. In blockchain, the valid transaction is added to the block after the validation process using consensus protocols. In addition, the ledger of each node is updated, and this process is publicly visible. Hence, a third party cannot add false transactions to the ledger. This visibility ensures the transparency and security of blockchain [78].

#### 2.2.4. Resistance to Attacks

**Figure 4:**
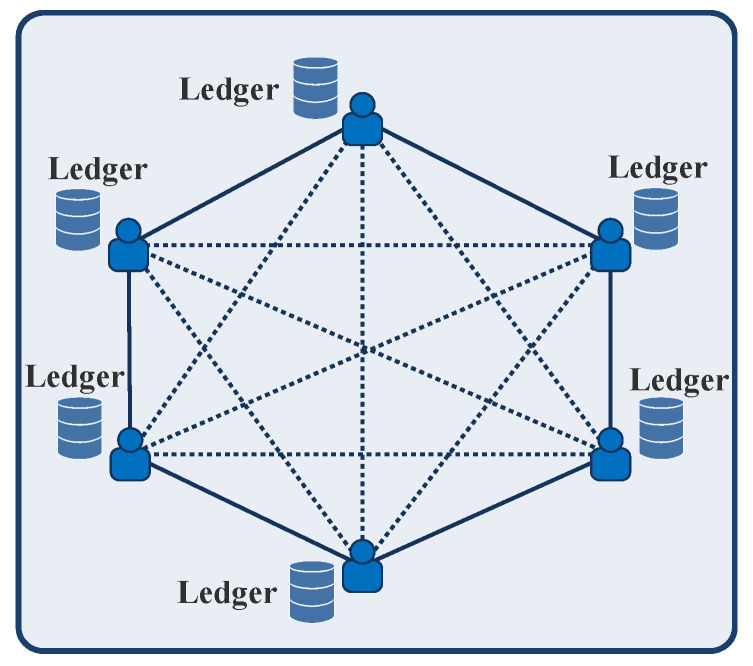
An example of blockchain network.

All of the nodes in the blockchain network hold identical copies of the ledger records, as shown in Figure 4, and update when the transaction is valid. Hence, blockchain is resistant to attacks and information leakage [12]. This feature of blockchain contributes to the network’s resilience and data integrity.

### 2.3. Types of Blockchain

According to different types of applications, blockchains are classified into three main categories [12], namely public blockchain, private blockchain, and consortium blockchain.

#### 2.3.1. Public Blockchains

**Figure 5:**
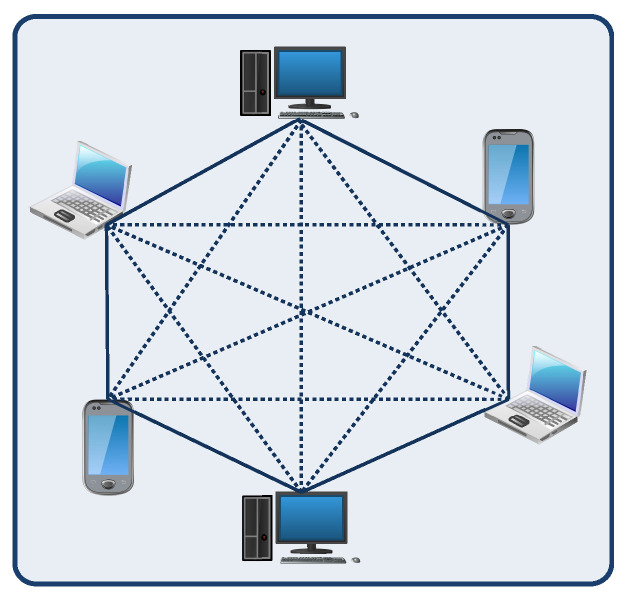
Public blockchain [79].

A public blockchain is fully decentralized, where any participant can participate in creating new blocks and can access the content of a blockchain. Figure 5 shows the structure of a public blockchain. In a public blockchain, anyone can keep a copy of blockchain and participate in the validation process of new blocks. Such a type of blockchain is also known as a permissionless blockchain because anyone can join without any permission. A public blockchain network consists of a large number of nodes; hence, it is resistant to malicious attacks. Additionally, each transaction has some processing fees as an incentive for a user who participates in the validating process. This makes the public blockchain more transparent and secure. Examples of public blockchains are cryptocurrency networks such as Bitcoin and Ethereum.

#### 2.3.2. Private Blockchains

**Figure 6:**
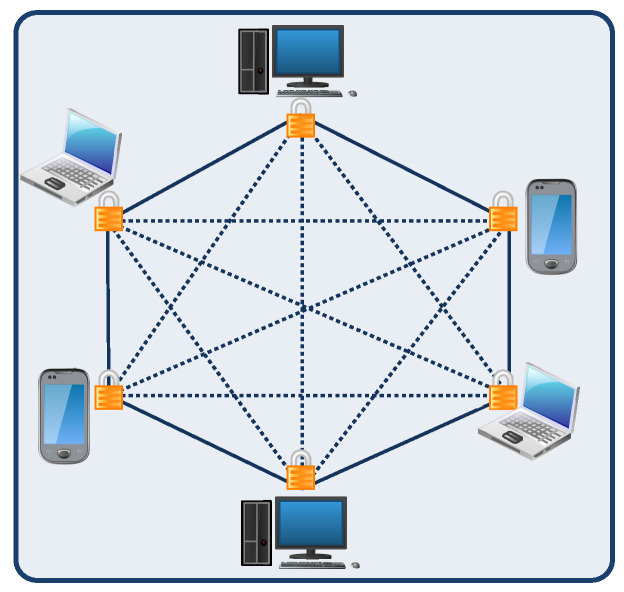
Private blockchain [79].

A private blockchain is also known as a permissioned blockchain, where every node is a member of a single organization or institute. Figure 6 illustrates the structure of a private blockchain. In a private blockchain, an authority can access the content of blockchain and permit other users to access the content. There are no transaction processing fees in a private blockchain, which is similar to that of a centralized system; however, it is cryptographically secure.

#### 2.3.3. Consortium Blockchains

**Figure 7:**
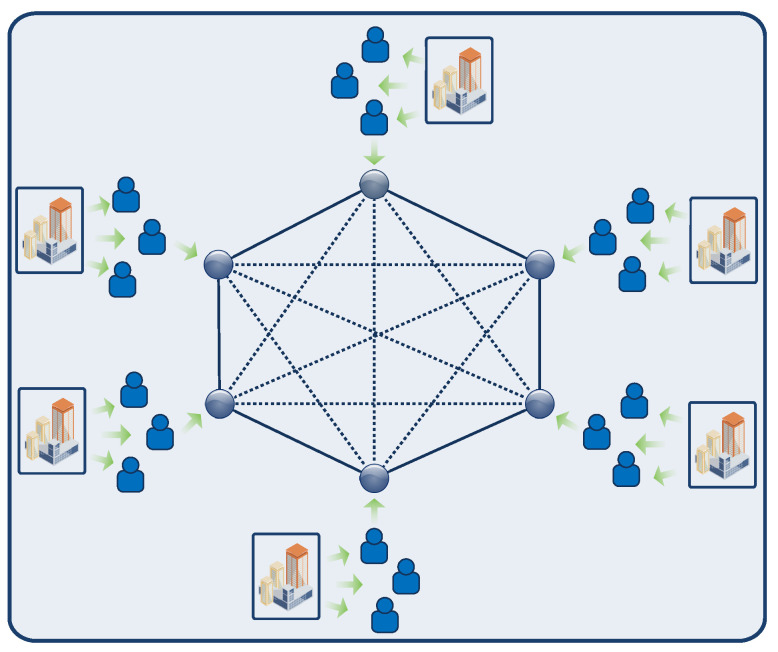
Consortium blockchain [79].

A consortium blockchain is a special type of private blockchain where a selected number of participants from multiple organizations can participate in the consensus process. Figure 7 depicts the structure of a consortium blockchain. A consortium blockchain helps to maintain transparency between the involved organizations. Similar to a private blockchain, there are no transaction processing fees; hence, it has a lower cost. A consortium blockchain is partially decentralized or tamper-proof. An example of a consortium blockchain is Hyperledger.

## 3. Overview of Quantum Key Distribution

**Figure 8:**
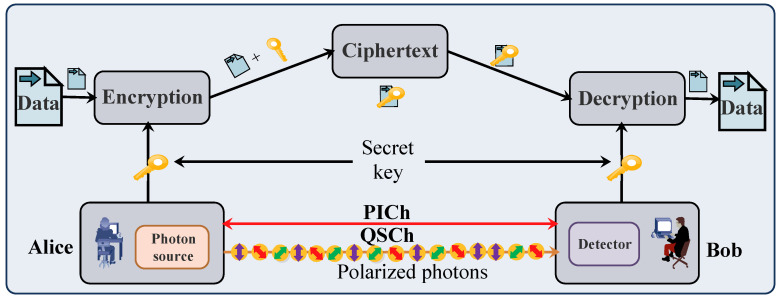
QKD process [47].

This section provides an overview of QKD technology for secure communication. QKD relies on the fundamental principles of quantum mechanics, namely the Heisenberg’s uncertainty principle and the quantum no-cloning theorem [48,55,56,80]. QKD establishes a secure connection between the end-users by generating and distributing secret keys over an insecure channel. A QKD protocol generates a secret key between the end-users and ensures security against eavesdropping. Several QKD protocols are designed for secret key generation, discussed in [54,81,82,83,84,85,86]. The most widely used QKD protocol is the BB84 protocol proposed in 1984 [48]. Additionally, for secret key generation and distribution, QKD requires two channels, namely QSCh and a public interaction channel (PICh), as shown in Figure 8 [47]. QSCh sends quantum bits (qubits), i.e., encoded polarization photons, between the end-users. PICh is used to transmit the measuring basis of qubits and verify the secret keys using post-processing methods.

### QKD Process

Figure 8 explains the QKD process for generating secret keys between Alice (sender) and Bob (receiver). The following steps describe the process of a QKD system with the BB84 QKD protocol [47].

- Alice generates a random string of bits, and, for each bit, she will randomly choose a basis: either rectilinear (two polarization states, i.e., $0^{\circ }$ or $90^{\circ }$) or diagonal (two polarization states, i.e., $+45^{\circ }$ or $-45^{\circ }$) with their polarization states. The random string of bits encoded with these polarization states is known as qubits. Alice then sends qubits to Bob through QSCh.
- Bob receives the qubits from Alice, measures the received qubits with one of the randomly selected measuring bases, and obtains a string of all received qubits from the measurement result.
- Alice and Bob exchange their measurement bases through PICh and compare them. After comparison, the qubits with different measuring bases are discarded. The remaining qubits that correspond to the same measuring bases are decoded into a string of binary bits known as a *sifted key* [49,87].
- A random substring of a sifted key is exchanged and compared for parameter estimation and error correction between Alice and Bob via PICh.
- Privacy amplification and authentication are performed, which reduces the information of remaining bits against the eavesdropping and generates a new shorter key known as a *secret key* [88,89].
- After secret key generation, the encryption process starts. In this, the generated secret key encrypts the information transmitted by Alice and converts the information into ciphertext using a one-time pad encryption [90] and symmetric encryption algorithm, i.e., the advanced encryption standard (AES) [91]. Now, Bob uses the same secret key for decryption, i.e., converting the ciphertext into the original information. In this way, Alice and Bob securely communicate with each other using QKD [47].

## 4. Quantum-Secured Blockchain

This section describes the quantum-secured blockchain technology along with its underlying process. Blockchain technology is strong enough to provide security within the blockchain network between the nodes by leveraging asymmetric cryptography and hashing algorithms. Asymmetric cryptography generates a pair of keys to provide security between the nodes and authenticate transactions by generating a digital signature. The most widely used digital signature schemes are Rivest, Shamir, Adleman (RSA) [92], or elliptic curve cryptography [46]. Hashing algorithms also play a crucial role in providing security by hashing the transaction data and linking blocks of a blockchain by generating block hash values. However, the security of both asymmetric cryptography and hash algorithms relies on the computational complexity of certain mathematical functions that quantum computers can easily attack shortly [45,93]. Hence, blockchain will release all of its security features and become insecure. If quantum attack-aware schemes are not designed to enhance blockchain security, then the existing and future blockchain networks will become vulnerable and put blockchain at risk.

Post-quantum cryptography schemes [46] were proposed to overcome the blockchain security problem. However, currently, their security is questionable. Hence, they do not provide guaranteed security against threats. The most prominent way to provide complete security in blockchain against quantum attacks is QKD. The security of QKD relies on the fundamental laws of quantum mechanics [58]. QKD generates and distributes random secret keys between the authenticated users in the network using the QKD protocol through QSCh and PICh to encrypt confidential information. Hence, there is a huge research interest in protecting the blockchain network against quantum attacks by integrating QKD into blockchain [94]. A quantum-secured blockchain platform was developed and experimentally demonstrated, which uses QKD for authentication and the original BFT consensus protocol for validation [45]. The security of the quantum-safe blockchain is practically realizable and scalable for different government and commercial services. However, a major drawback of the proposed quantum-secured blockchain is the use of a consensus protocol. The limitation of the BFT consensus protocol is that, if a large number of non-operational nodes are present in the blockchain network, it becomes data-intensive. Hence, a new quantum-secured consensus protocol was designed to limit the problem of the traditional consensus protocol in [63]. However, not many protocols have been implemented to improve the security of blockchain networks using QKD. Therefore, further research is urgently needed to design secure consensus protocols using quantum technologies.

### 4.1. Process of Quantum Blockchain

**Figure 9:**
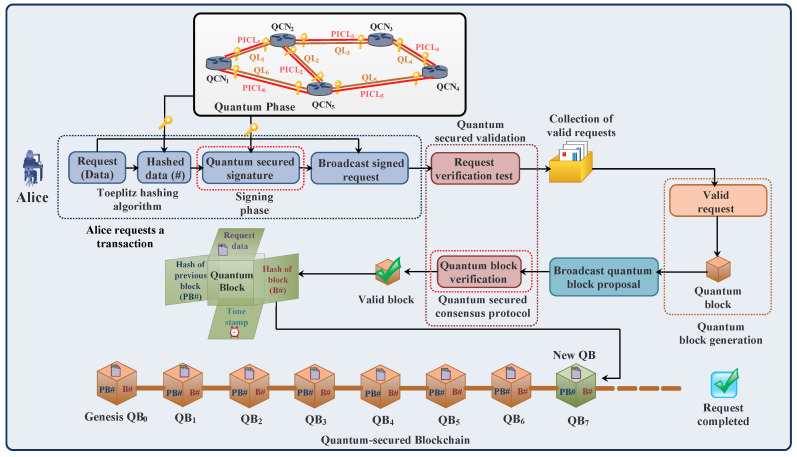
Process of quantum-secured blockchain [45,63].

This subsection discusses the process of quantum blockchain. In quantum blockchain, the QKD technique is used to generate and distribute secret keys and provide authentication, which makes blockchain networks robust against the attacking capabilities of quantum computers [43,95]. Quantum blockchain uses the same components as the traditional blockchain, discussed in Section 2.1.1. However, a major difference is that, instead of conventional cryptography and hashing algorithms, it utilizes quantum techniques to secure the network against security breaches. Figure 9 shows the workflow of a quantum blockchain [45,63]. The workflow consists of the quantum phase, transaction proposal phase, transaction validation phase, and quantum block proposal and validation phase. A detailed description of the phases is discussed below.

#### 4.1.1. Quantum Phase

A quantum phase consists of a QKD network [96,97,98,99,100,101], as shown in Figure 9. In this phase, random secret keys between the two authenticated users in the network are generated using QKD protocols, such as BB84 [48,54] and others [81,82,83,84,85,86,102,103,104,105,106,107], through QSCh and PICh, discussed in Section 3. The generated secret keys are then used for encryption and authentication.

#### 4.1.2. Transaction Proposal Phase

In the transaction proposal phase, Alice requests a transaction and hashed data by using hashing algorithms for encryption, as shown in Figure 9. The most widely used scheme is Toeplitz hashing [108], in which a Toeplitz matrix is generated by shared random keys between the sender and receiver. This scheme, along with one-time pad encryption, helps in transferring transaction data securely. The generated secret keys using QKD in the quantum phase are used in generating a quantum-secured signature to sign a transaction in a signing phase. After the signing phase, the transaction data and the signature are broadcasted to the nodes in the quantum blockchain network.

#### 4.1.3. Transaction Validation Phase

In this phase, upon receiving the transaction data and signature, the blockchain participants perform a specific test, detailed in [63], to validate the transaction. After validation, only the valid transactions are collected in a block of valid requests, as shown in Figure 9.

#### 4.1.4. Quantum Block Proposal and Validation Phase

After the transaction validation phase, the quantum block (QB) of valid requests is created and broadcasted to peer nodes in the quantum blockchain network for validation. The QB is validated using quantum-secured consensus protocols consisting of proposing, voting, and decision phases, as explained in [63]. When the QB is validated, it is then added to the quantum blockchain to form a quantum-secured blockchain. After that, the ledger of each node in the quantum blockchain network is updated, and the transaction is securely received.

## 5. Security in Optical Networks Using Quantum-Secured Blockchain

**Figure 10:**
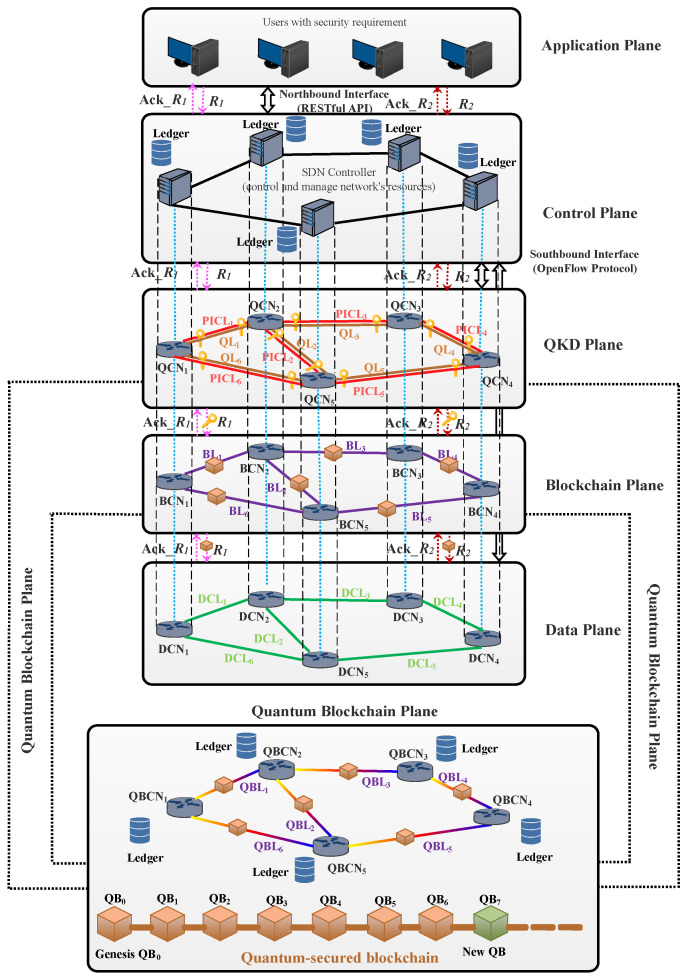
Distributed quantum-secured blockchain-based optical network architecture [23,61,80].

In this section, the article explains the proposed distributed quantum-secured blockchain-based optical network architecture for future research to enhance the security of optical networks. The readers interested in QKD and different architecture of QKD-secured optical networks studies are encouraged to refer to the literature [49,55,57,58,61,80,109,110]. Integrating QKD with blockchain increases the security of the overall optical networks [45]. Blockchain, along with QKD, includes all of the security and privacy characteristics that are essential for existing and future optical networks. The distributed quantum-secured blockchain architecture for optical networks is presented in Figure 10.

### 5.1. Distributed Quantum-Secured Blockchain Optical Network Architecture

The architecture consists of five planes: an application plane, control plane, QKD plane [55,61,80], blockchain plane [14], and data plane. The description of each plane with an example is discussed in this subsection and shown in Figure 10.

#### 5.1.1. Application Plane

The application plane generates lightpath requests of different security levels as per user requirements and sends them to the control plane for further processing. The acceptance/rejection status of each lightpath request is received at the application plane.

#### 5.1.2. Control Plane

In this distributed network architecture, the control plane is implemented by using SDN controllers. The SDN controller efficiently controls and manages the network’s resources. After the generation of lightpath requests from the application plane, the control plane alerts the QKD plane, blockchain plane, and data plane. The control plane allocates resources for QSCh and PICh in the QKD plane, and blockchain channel (BCCh) in the blockchain plane, i.e., for the quantum blockchain channel (QBCh) in the quantum blockchain plane, and TDCh in the data plane.

#### 5.1.3. QKD Plane

The QKD plane is implemented using the QKD protocol, such as the BB84 protocol and others [55], for secret key generation between the end users, as discussed in Section 3. The generated secret keys through QSCh and PICh are used for blockchain security in the blockchain plane. In addition, the weak quantum signals have a significantly shorter transmission reach; therefore, several intermediate trusted repeater nodes (TRNs) need to be placed to achieve long-distance secure communication.

#### 5.1.4. Blockchain Plane

The blockchain plane generates different blocks of chain using secure quantum technology, as discussed in Section 4. The generated blockchain facilitates the process of recording and tracking requests without the need of any single centralized trusted authority. This plane helps in maintaining a ledger at each node in the control plane. Hence, it is tamper-resistant.

#### 5.1.5. Data Plane

The data plane serves the lightpath requests in a similar way to data transmission in conventional optical networks; however, with added security. After each secure transmission, a ledger of all nodes in the quantum-secured blockchain optical network is updated.

### 5.2. Description of the Process and the Architecture with an Example

This subsection discusses the process and the architecture of the presented work with an example. Figure 9 and Figure 10 illustrate the process of quantum blockchain and the architecture of a quantum-secured blockchain-based optical network, respectively. In this architecture, different protocols are used to establish communication among five planes. The OpenFlow protocol is used to implement the southbound interface for the control plane (consisting of an SDN controller) and QKD/blockchain/data plane [80,111]. For the northbound interface, the RESTful application program interface (API) is used between the control plane and application plane [61,80], as shown in Figure 10. For ease of understanding, let us consider that Alice requests a transaction, and the application plane generates a lightpath request *R_1_* from the source node *1* to the destination node *2* with the security requirement in the network. Upon receiving a lightpath request *R_1_* from the application plane, the distributed control plane alerts the QKD plane to generate and distribute secret keys between the nodes in the network via quantum links (QLs) and public interaction channel links (PICLs) (according to the process discussed in Section 3). The control plane then transmits control to the blockchain plane to securely transfer the blocks of information using the generated secret key in QKD plane through blockchain links (BLs) following the process with different phases discussed in Section 4.1. The combination of the QKD plane and blockchain plane of the network architecture is known as the quantum blockchain plane, where the information is in the form of quantum blocks transferred through quantum blockchain links (QBLs). In the end, the data plane provides an end-to-end transport of lightpath requests between the data communication nodes (DCNs) through data channel links (DCLs) in the network. After lightpath request establishment, the data plane acknowledges the control plane. The control plane, after acknowledgment, updates the network’s resources, the ledger of each node, and the status of lightpath requests generated from the application plane. In this way, a lightpath request *R_1_* is securely established in the network using quantum-secured blockchain technology.

## 6. Research Challenges, Opportunities, and Directions

### 6.1. Research Challenges and Opportunities

Blockchain is an emerging technology used in various applications, including but not limited to the Internet of Things (IoT), wireless communication networks, healthcare networks, financial systems, supply chains, and voting systems. However, the evolution of quantum computers will easily break the security of blockchain technology and destroy the existing and next-generation blockchain networks. Inspired by recent advancements in quantum technology, researchers and developers are increasing their interest in combining one of the most promising quantum communication techniques, i.e., QKD, with blockchain to secure blockchain against quantum attacks. Integrating quantum with blockchain introduces various challenges that need to be addressed in the near future. In this subsection, the article discusses the research challenges and opportunities for future research in quantum-secured blockchain technology for optical networks.

In quantum-secured blockchain, the level of security, transaction speed, and network scalability are ultimately determined by the consensus method. A consensus method is a process that enables every peer in the blockchain network to agree on the present state of the distributed ledger. However, not many quantum-based consensus protocols have been designed. Hence, significant research efforts are required to develop new quantum-secured consensus protocols to enhance blockchain security in optical networks. In addition to this, to build trust on the blockchain, digital signatures serve as basic cryptographic proof systems. However, in quantum blockchain, not much research has been conducted on designing quantum-based signature schemes that can help in establishing trust in the quantum blockchain network. Hence, different quantum-secured signature schemes need to be designed for the signing phase in quantum-secured blockchain because the conventional signature schemes are not strong enough for authentication, as it is based on elliptic curve cryptography. Moreover, to check the validity of the transaction, participants in quantum-secured blockchain perform a verification test. Hence, various verification schemes need to be proposed to make quantum blockchain more secure and reliable for quantum-secured blockchain network participants. Furthermore, deploying cost-effective quantum-based blockchain optical networks is the biggest challenge from the perspective of network architecture. Hence, efforts are also required to develop cost-efficient solutions for quantum-assisted blockchain-based optical networks. Moreover, from a networking point of view, one of the most critical challenges in the quantum-secured blockchain optical networks is resilience against node/link failure, which can affect the communication between nodes in the network. Therefore, new survivability schemes need to be developed for such optical networks.

Apart from these, for security improvement, the quantum-secured blockchain can be deployed for a variety of blockchain-based applications, such as secure IoT networks and personal information, logistic and supply chain tracking, financial exchanges, government services, and many more in the future.

### 6.2. Research Directions

This subsection discusses the research directions in the area of quantum-secured blockchain technology. In the area of communication and networking, artificial intelligence (AI), machine learning (ML), deep learning (DL), and reinforcement learning (RL) have been used as effective solutions to address various problems and challenges. AI/ML has the capability to take decisions and automate and optimize the system for better performance. For an improved security, speed, and scalability, AI/ML can help to construct an intelligent system on the quantum-secured blockchain. Additionally, recent advances in AI/ML, such as DL and RL, can be exploited to propose a secure and robust consensus in quantum-secured blockchain. AI/ML plays an important role in providing protection against node/link failures in quantum blockchain-based optical networks with confidentiality and privacy. A new combination of quantum-secured blockchain and AI/ML techniques will be able to build more robust and trusted optical networks against various security breaches. However, such a combination of security and intelligence is not currently developed and also might face various challenges in this domain. Hence, efforts are needed to combine quantum-assisted blockchain with AI/ML/DL/RL and design more secure, trusted, and intelligent optical networks.

## 7. Conclusions

Vulnerabilities affect optical network infrastructure and services developed for highly secure bandwidth-hungry applications such as military, finance utilities, and other government services, and cause a large amount of data and revenue loss. Hence, blockchain technology has been adopted to securely transmit data between untrusted nodes in optical networks. However, blockchain will be vulnerable once quantum computers become easily available. Hence, quantum technology-based solutions can provide opportunities to secure blockchain networks.

This article provided a broad view of the quantum-secured blockchain technology and focused on the current research efforts in developing secure and robust optical networks. This article began with a brief overview of blockchain technology, which is a distributed database with verifiable and immutable records of transaction. In addition to this, the article explained the concept of one of the most promising applications of quantum communication, i.e., QKD with its secret key generation process using the BB84 protocol. The reasons behind integrating QKD into the blockchain to design a quantum-secured blockchain and using quantum-secured blockchain in optical networks were discussed in this article. A general distributed quantum-secured blockchain optical network architecture was presented. The architecture describes the operation of each plane to develop secure and trusted optical networks for highly secure applications against various attacks in future research. At the end, the article highlighted the research challenges that need to be explored in the near future and provided research directions for the researchers and developers. This article raised interest towards enhancing security in optical networks and various blockchain-based applications using quantum-secured blockchain.

## Data Availability

Not applicable.

## Abbreviations

The following abbreviations are used in this manuscript:

AI	Artificial Intelligence
BB84	Bennett and Brassard-84
BCCh	Blockchain Channel
BFT	Byzantine Fault Tolerance
DCLs	Data Communication Links
DCNs	Data Communication Nodes
DL	Deep Learning
DPoS	Delegated Proof-of-Stake
GDP	Gross Domestic Product
GHZ	Greenberger–Horne–Zeilinger
IoT	Internet of Things
ML	Machine Learning
PICh	Public Interaction Channel
PICLs	Public Interaction Channel Links
PoS	Proof-of-Stake
QBs	Quantum Blocks
QBCh	Quantum Blockchain Channel
QBLs	Quantum Blockchain Channel Links
QKD	Quantum Key Distribution
QLs	Quantum Links
QoT	Quality of Transmission
QSCh	Quantum Signal Channel
RL	Reinforcement Learning
RSA	Rivest, Shamir, Aldeman
SDN	Software-defined Network
SDON	Software-defined Optical Network
SLA	Service Level Agreement
TDCh	Traditional Data Channel
TRNs	Trusted Repeater Nodes
VONs	Virtual Optical Networks

## References

[1] Skorin-Kapov N., Furdek M., Zsigmond S., Wosinska L. (2016). Physical-layer security in evolving optical networks. IEEE Commun. Mag..

[2] Furdek M., Skorin-Kapov N., Zsigmond S., Wosinska L. Vulnerabilities and security issues in optical networks. Proceedings of the 16th International Conference on Transparent Optical Networks (ICTON).

[3] Rawat D.B., Reddy S.R. (2016). Software defined networking architecture, security and energy efficiency: A survey. IEEE Commun. Surv. Tuts..

[4] Hussain M., Shah N., Amin R., Alshamrani S.S., Alotaibi A., Raza S.M. (2022). Software-Defined Networking: Categories, Analysis, and Future Directions. Sensors.

[5] Alvizu R., Maier G., Kukreja N., Pattavina A., Morro R., Capello A., Cavazzoni C. (2017). Comprehensive survey on T-SDN: Software-defined networking for transport networks. IEEE Commun. Surv. Tuts..

[6] Gringeri S., Bitar N., Xia T.J. (2013). Extending software defined network principles to include optical transport. IEEE Commun. Mag..

[7] Ndiaye M., Hancke G.P., Abu-Mahfouz A.M. (2017). Software defined networking for improved wireless sensor network management: A survey. Sensors.

[8] Urrea C., Benítez D. (2021). Software-defined networking solutions, architecture and controllers for the industrial internet of things: A review. Sensors.

[9] Kou S., Yang H., Zheng H., Bai W., Zhang J., Wu Y. Blockchain Mechanism Based on Enhancing Consensus for Trusted Optical Networks. Proceedings of the Asia Communications and Photonics Conference (ACP).

[10] Luo G., Han Z., Lu L., Hussain M.J. Real-time and passive wormhole detection for wireless sensor networks. Proceedings of the 20th IEEE International Conference on Parallel and Distributed Systems (ICPADS).

[11] Yu H., Kaminsky M., Gibbons P.B., Flaxman A.D. (2008). Sybilguard: Defending against sybil attacks via social networks. IEEE/ACM Trans. Netwo..

[12] Ali M.S., Vecchio M., Pincheira M., Dolui K., Antonelli F., Rehmani M.H. (2018). Applications of blockchains in the Internet of Things: A comprehensive survey. IEEE Commun. Surv. Tutor..

[13] Krichen M., Ammi M., Mihoub A., Almutiq M. (2022). Blockchain for modern applications: A survey. Sensors.

[14] Aggarwal S., Chaudhary R., Aujla G.S., Kumar N., Choo K.K.R., Zomaya A.Y. (2019). Blockchain for smart communities: Applications, challenges and opportunities. J. Netw. Comput. Appl..

[15] Kumar S., Rathore R.S., Mahmud M., Kaiwartya O., Lloret J. (2022). BEST—Blockchain-Enabled Secure and Trusted Public Emergency Services for Smart Cities Environment. Sensors.

[16] Rathod T., Jadav N.K., Alshehri M.D., Tanwar S., Sharma R., Felseghi R.A., Raboaca M.S. (2022). Blockchain for Future Wireless Networks: A Decade Survey. Sensors.

[17] Rathore H., Mohamed A., Guizani M. (2020). A survey of blockchain enabled cyber-physical systems. Sensors.

[18] Deepa N., Pham Q.-V., Nguyen D.C., Bhattacharya S., Prabadevi B., Gadekallu T.R., Maddikunta P.K.R., Fang F., Pathirana P.N. (2022). A survey on blockchain for big data: Approaches, opportunities, and future directions. Future Gener. Comput. Syst..

[19] Casino F., Dasaklis T.K., Patsakis C. (2019). A systematic literature review of blockchain-based applications: Current status, classification and open issues. Telemat. Inform..

[20] Li X., Jiang P., Chen T., Luo X., Wen Q. (2020). A survey on the security of blockchain systems. Future Gener. Comput. Syst..

[21] Liu Z., Luong N.C., Wang W., Niyato D., Wang P., Liang Y.-C., Kim D.I. (2019). A survey on blockchain: A game theoretical perspective. IEEE Access.

[22] Yang R., Yu F.R., Si P., Yang Z., Zhang Y. (2019). Integrated blockchain and edge computing systems: A survey, some research issues and challenges. IEEE Commun. Surv. Tutor..

[23] Yang H., Liang Y., Yao Q., Guo S., Yu A., Zhang J. (2019). Blockchain-based secure distributed control for software defined optical networking. IEEE China Commun..

[24] Ismail L., Materwala H. (2019). A review of blockchain architecture and consensus protocols: Use cases, challenges, and solutions. Symmetry.

[25] Bodkhe U., Tanwar S., Parekh K., Khanpara P., Tyagi S., Kumar N., Alazab M. (2020). Blockchain for industry 4.0: A comprehensive review. IEEE Access.

[26] Liu G., Fan N., Wu C.Q., Zou X. (2022). On a blockchain-based security scheme for defense against malicious nodes in vehicular ad-hoc networks. Sensors.

[27] Palaiokrassas G., Skoufis P., Voutyras O., Kawasaki T., Gallissot M., Azzabi R., Tsuge A., Litke A., Okoshi T., Nakazawa J. (2021). Combining Blockchains, Smart Contracts, and Complex Sensors Management Platform for Hyper-Connected SmartCities: An IoT Data Marketplace Use Case. Computers.

[28] McGhin T., Choo K.K.R., Liu C.Z., He D. (2019). Blockchain in healthcare applications: Research challenges and opportunities. J. Netw. Comput. Appl..

[29] Taralunga D.D., Florea B.C. (2021). A blockchain-enabled framework for mhealth systems. Sensors.

[30] Abbas A., Alroobaea R., Krichen M., Rubaiee S., Vimal S., Almansour F.M. (2021). Blockchain-assisted secured data management framework for health information analysis based on Internet of Medical Things. Pers. Ubiquitous Comput..

[31] Agarwal U., Rishiwal V., Tanwar S., Chaudhary R., Sharma G., Bokoro P.N., Sharma R. (2022). Blockchain Technology for Secure Supply Chain Management: A Comprehensive Review. IEEE Access.

[32] Zafar S., Hassan S.F.U., Mohammad A.S., Al-Ahmadi A.A., Ullah N. (2022). Implementation of a Distributed Framework for Permissioned Blockchain-Based Secure Automotive Supply Chain Management. Sensors.

[33] Cai Z., Liu S., Han Z., Wang R., Huang Y. (2021). A Quantum Blind Multi-Signature Method for the Industrial Blockchain. Entropy.

[34] Nakamoto S. (2008). Bitcoin: A Peer-to-Peer Electronic Cash System. https://bitcoin.org/bitcoin.pdf.

[35] Swan M. (2015). Blockchain: Blueprint for a New Economy.

[36] Tschorsch F., Scheuermann B. (2016). Bitcoin and beyond: A technical survey on decentralized digital currencies. IEEE Commun. Surv. Tutor..

[37] Marr B. (2016). How Blockchain Technology Could Change The World. Forbes.

[38] Fichera S., Sgambelluri A., Giorgetti A., Cugini F., Paolucci F. Blockchain-anchored Failure Responsibility Management in Disaggregated Optical Networks. Proceedings of the Optical Fiber Communications Conference and Exhibition (OFC).

[39] Fichera S., Sgambelluri A., Paolucci F., Giorgetti A., Sambo N., Castoldi P., Cugini F. (2021). Blockchain-anchored disaggregated optical networks. IEEE J. Light. Technol..

[40] Alemany P., Vilalta R., Muñoz R., Martínez R., Casellas R. Managing network slicing resources using blockchain in a multi-domain software defined optical network scenario. Proceedings of the European Conference on Optical Communications (ECOC).

[41] Ding S., Shen G., Pan K.X., Bose S.K., Zhang Q., Mukherjee B. (2020). Blockchain-assisted spectrum trading between elastic virtual optical networks. IEEE Netw..

[42] Yang H., Wu Y., Zhang J., Zheng H., Ji Y., Lee Y. BlockONet: Blockchain-based trusted cloud radio over optical fiber network for 5G fronthaul. Proceedings of the Optical Fiber Communications Conference and Exposition (OFC).

[43] Debnath S., Linke N.M., Figgatt C., Landsman K.A., Wright K., Monroe C. (2016). Demonstration of a small programmable quantum computer with atomic qubits. Nature.

[44] Raussendorf R., Briegel H.J. (2001). A one-way quantum computer. Phys. Rev. Lett..

[45] Kiktenko E.O., Pozhar N.O., Anufriev M.N., Trushechkin A.S., Yunusov R.R., Kurochkin Y.V., Lvovsky A., Fedorov A. (2018). Quantum-secured blockchain. Quantum Sci. Technol..

[46] Fernández-Caramés T.M., Fraga-Lamas P. (2020). Towards Post-Quantum Blockchain: A Review on Blockchain Cryptography Resistant to Quantum Computing Attacks. IEEE Access.

[47] Mailloux L.O., Grimaila M.R., Hodson D.D., Baumgartner G., McLaughlin C. (2015). Performance evaluations of quantum key distribution system architectures. IEEE Secur. Priv..

[48] Bennett C., Brassard G. Quantum cryptography: Public key distribution and coin tossing. Proceedings of the International Conference on Computers, Systems & Signal Processing.

[49] Zhao Y., Cao Y., Yu X., Zhang J., Morozov O.G. (2019). Quantum Key Distribution (QKD) over Software-Defined Optical Networks. Quantum Cryptography in Advanced Networks.

[50] Heisenberg W., Wheeler J.A., Zurek W.H. (1927). The Physical Content of Quantum Kinematics and Mechanics. Quantum Theory and Measurement.

[51] Heisenberg W. (1930). Physical Principles of the Quantum Theory.

[52] Wootters W.K., Zurek W.H. (1982). A single quantum cannot be cloned. Nature.

[53] Adu-Kyere A., Nigussie E., Isoaho J. (2022). Quantum Key Distribution: Modeling and Simulation through BB84 Protocol Using Python3. Sensors.

[54] Bennett C.H., Brassard G. (2014). Quantum cryptography: Public key distribution and coin tossing. Theor. Comput. Sci..

[55] Sharma P., Agrawal A., Bhatia V., Prakash S., Mishra A.K. (2021). Quantum key distribution secured optical networks: A survey. IEEE Open J. Commun. Soc..

[56] Zhang Q., Xu F., Chen Y.A., Peng C.Z., Pan J.W. (2018). Large scale quantum key distribution: Challenges and solutions. Opt. Express.

[57] Mafu M., Senekane M., Gnatyuk S. (2018). Security of Quantum Key Distribution Protocols. Advanced Technologies of Quantum Key Distribution.

[58] Lo H.K., Curty M., Tamaki K. (2014). Secure quantum key distribution. Nat. Photon..

[59] Diamanti E., Lo H.K., Qi B., Yuan Z. (2016). Practical challenges in quantum key distribution. NPJ Quantum Inf..

[60] Xu F., Ma X., Zhang Q., Lo H.-K., Pan J.-W. (2020). Secure quantum key distribution with realistic devices. Rev. Mod. Phys..

[61] Zhao Y., Cao Y., Wang W., Wang H., Yu X., Zhang J., Tornatore M., Wu Y., Mukherjee B. (2018). Resource allocation in optical networks secured by quantum key distribution. IEEE Commun. Mag..

[62] Wang W., Yu Y., Du L. (2022). Quantum blockchain based on asymmetric quantum encryption and a stake vote consensus algorithm. Sci. Rep..

[63] Sun X., Sopek M., Wang Q., Kulicki P. (2019). Towards Quantum-Secured Permissioned Blockchain: Signature, Consensus, and Logic. Entropy.

[64] Rajan D., Visser M. (2019). Quantum blockchain using entanglement in time. Quantum Rep..

[65] Aharonov Y., Popescu S., Tollaksen J., Vaidman L. (2009). Multiple-time states and multiple-time measurements in quantum mechanics. Phys. Rev. A.

[66] Brukner C., Taylor S., Cheung S., Vedral V. (2004). Quantum entanglement in time. arXiv.

[67] Ringbauer M., Costa F., Goggin M.E., White A.G., Fedrizzi A. (2018). Multi-time quantum correlations with no spatial analog. NPJ Quantum Inf..

[68] Iovane G. (2021). MuReQua Chain: Multiscale Relativistic Quantum Blockchain. IEEE Access.

[69] Coladangelo A., Sattath O. (2020). A quantum money solution to the blockchain scalability problem. Quantum.

[70] Abd El-Latif A.A., Abd-El-Atty B., Mehmood I., Muhammad K., Venegas-Andraca S.E., Peng J. (2021). Quantum-inspired blockchain-based cybersecurity: Securing smart edge utilities in IoT-based smart cities. Inf. Process. Manag..

[71] Gao Y.-L., Chen X.-B., Xu G., Yuan K.-G., Liu W., Yang Y.-X. (2020). A novel quantum blockchain scheme base on quantum entanglement and DPoS. Quantum Inf. Process..

[72] Qu Z., Zhang Z., Zheng M. (2022). A quantum blockchain-enabled framework for secure private electronic medical records in Internet of Medical Things. Inf. Sci..

[73] Belotti M., Božić N., Pujolle G., Secci S. (2019). A Vademecum on Blockchain Technologies: When, Which, and How. IEEE Commun. Surv. Tuts..

[74] Park J.H., Park J.H. (2017). Blockchain security in cloud computing: Use cases, challenges, and solutions. Symmetry.

[75] Shi N. (2016). A new proof-of-work mechanism for bitcoin. Financ. Innov..

[76] Wang W., Hoang D.T., Hu P., Xiong Z., Niyato D., Wang P., Wen Y., Kim D.I. (2019). A survey on consensus mechanisms and mining strategy management in blockchain networks. IEEE Access.

[77] Rahman A.R., Islam M.J., Rahman Z., Reza M.M., Anwar A., Mahmud M.A.P., Nasir M.K., Noor R.M. (2020). Distb-condo: Distributed blockchain-based IoT-SDN model for smart condominium. IEEE Access.

[78] Sengupta J., Ruj S., Bit S.D. (2020). A comprehensive survey on attacks, security issues and blockchain solutions for IoT and IIoT. J. Netw. Comput..

[79] Rahman A., Montieri A., Kundu D., Karim M.R., Islam M.J., Umme S., Nascita A., Pescapé A. (2022). On the Integration of Blockchain and SDN: Overview, Applications, and Future Perspectives. J. Netw. Syst. Manag..

[80] Cao Y., Zhao Y., Yu X., Wu Y. (2017). Resource assignment strategy in optical networks integrated with quantum key distribution. J. Opt. Commun. Netw..

[81] Scarani V., Bechmann-Pasquinucci H., Cerf N.J., Dušek M., Lütkenhaus N., Peev M. (2009). The security of practical quantum key distribution. Rev. Mod. Phys..

[82] Ekert A.K. (1991). Quantum cryptography based on Bell’s theorem. Phys. Rev. Lett..

[83] Inoue K., Waks E., Yamamoto Y. (2002). Differential phase shift quantum key distribution. Phys. Rev. Lett..

[84] Inoue K., Waks E., Yamamoto Y. (2003). Differential-phase-shift quantum key distribution using coherent light. Phys. Rev. A.

[85] Bechmann-Pasquinucci H., Gisin N. (1999). Incoherent and coherent eavesdropping in the six-state protocol of quantum cryptography. Phys. Rev. A.

[86] Bennett C.H., Brassard G., Mermin N.D. (1992). Quantum cryptography without Bell’s theorem. Phys. Rev. Lett..

[87] Cao Y., Zhao Y., Wu Y., Yu X., Zhang J. (2018). Time-scheduled quantum key distribution (QKD) over WDM networks. IEEE/OSA J. Lightw. Technol..

[88] Fung C.H.F., Ma X., Chau H. (2010). Practical issues in quantum-key-distribution postprocessing. Phys. Rev. A.

[89] Kiktenko E., Trushechkin A., Kurochkin Y., Fedorov A. (2016). Post-processing procedure for industrial quantum key distribution systems. J. Phys. Conf. Ser..

[90] Vernam G.S. (1926). Cipher printing telegraph systems: For secret wire and radio telegraphic communications. IEEE J. AIEE.

[91] Dworkin M., Barker E., Nechvatal J., Foti J., Bassham L., Roback E., JFD J. (2001). Advanced encryption standard (AES). Fed. Inf. Process. Stds. (NIST FIPS).

[92] Rivest R.L., Shamir A., Adleman L. (1978). A method for obtaining digital signatures and public-key cryptosystems. Commun. ACM.

[93] Schneier B. (1996). Applied Cryptography.

[94] Toudeh-Fallah F., Pistoia M., Kawakura Y., Moazzami N., Kramer D.H., Woodward R.I., Sysak G., John B., Amer O., Polychroniadou A.O. (2022). Paving the Way towards 800 Gbps Quantum-Secured Optical Channel Deployment in Mission-Critical Environments. arXiv.

[95] Fedorov A.K., Kiktenko E.O., Lvovsky A.I. (2018). Quantum computers put blockchain security at risk. Nature.

[96] Chip E., Alexander C., David P., Oleksiy P., John S., Henry Y., Donkor E.J., Pirich A.R., Brandt H.E. (2005). Current status of the DARPA quantum network. Proceedings of the Quantum Information and Computation III.

[97] Peev M., Pacher C., Alléaume R., Barreiro C., Bouda J., Boxleitner W., Debuisschert T., Diamanti E., Dianati M., Dynes J.F. (2009). The SECOQC quantum key distribution network in Vienna. New J. Phys..

[98] Sasaki M., Fujiwara M., Ishizuka H., Klaus W., Wakui K., Takeoka M., Miki S., Yamashita T., Wang Z., Tanaka A. (2011). Field test of quantum key distribution in the Tokyo QKD network. Opt. Express.

[99] Courtland R. (2016). China’s 2000-km quantum link is almost complete [News]. IEEE Spectr..

[100] Elliott C. (2002). Building the quantum network. N. J. Phys..

[101] Razavi M., Leverrier A., Ma X., Qi B., Yuan Z. (2019). Quantum key distribution and beyond: Introduction. JOSA B.

[102] Hwang W.-Y. (2003). Quantum key distribution with high loss: Toward global secure communication. Phys. Rev. Lett..

[103] Wang X.-B. (2005). Beating the photon-number-splitting attack in practical quantum cryptography. Phys. Rev. Lett..

[104] Lo H.-K. Quantum key distribution with vacua or dim pulses as decoy states. Proceedings of the International Symposium onInformation Theory.

[105] Lo H.-K., Curty M., Qi B. (2012). Measurement-device-independent quantum key distribution. Phys. Rev. Lett..

[106] Tang Y.-L., Yin H.-L., Zhao Q., Liu H., Sun X.-X., Huang M.-Q., Zhang W.-J., Chen S.-J., Zhang L., You L.-X. (2016). Measurement-device-independent quantum key distribution over untrustful metropolitan network. Phys. Rev. X.

[107] Lucamarini M., Yuan Z.L., Dynes J.F., Shields A.J. (2018). Overcoming the rate–distance limit of quantum key distribution without quantum repeaters. Nature.

[108] Krawczyk H. (1995). New hash functions for message authentication. Proceedings of the EUROCRYPT.

[109] Cao Y., Zhao Y., Colman-Meixner C., Yu X., Zhang J. (2017). Key on demand (KoD) for software-defined optical networks secured by quantum key distribution (QKD). Opt. Express.

[110] Cao Y., Zhao Y., Wang J., Yu X., Ma Z., Zhang J. (2019). SDQaaS: Software defined networking for quantum key distribution as a service. Opt. Express.

[111] Liang Y., Yang H., Yao Q., Guo S., Yu A., Zhang J. Blockchain-based efficient recovery for secure distributed control in software defined optical networks. Proceedings of the Optical Fiber Communications Conference and Exhibition (OFC).

